# MultiBacMam Bimolecular Fluorescence Complementation (BiFC) tool-kit identifies new small-molecule inhibitors of the CDK5-p25 protein-protein interaction (PPI)

**DOI:** 10.1038/s41598-018-23516-x

**Published:** 2018-03-23

**Authors:** Itxaso Bellón-Echeverría, Jean-Philippe Carralot, Andrea Araujo Del Rosario, Stephanie Kueng, Harald Mauser, Georg Schmid, Ralf Thoma, Imre Berger

**Affiliations:** 1 Chemical Biology, pRED Innovation Center Basel, F. Hoffmann-La Roche Ltd, Basel, Switzerland; 2 European Molecular Biology Laboratory, Grenoble Outstation, 6 rue Jules Horowitz, 38042 Grenoble, France; 3 Unit for Virus Host-Cell Interactions, Univ. Grenoble Alpes-EMBL-CNRS, 6 rue Jules Horowitz, 38042 Grenoble, France; 4 Global Technical Development, F. Hoffmann-La Roche Ltd, Basel, Switzerland; 5 BrisSynBio Centre and School of Biochemistry, Bristol University, Clifton, BS8 1TD United Kingdom

## Abstract

Protein-protein interactions (PPIs) are at the core of virtually all biological processes in cells. Consequently, targeting PPIs is emerging at the forefront of drug discovery. Cellular assays which closely recapitulate native conditions *in vivo* are instrumental to understand how small molecule drugs can modulate such interactions. We have integrated MultiBacMam, a baculovirus-based mammalian gene delivery tool we developed, with bimolecular fluorescence complementation (BiFC), giving rise to a highly efficient system for assay development, identification and characterization of PPI modulators. We used our system to analyze compounds impacting on CDK5-p25 PPI, which is implicated in numerous diseases including Alzheimer’s. We evaluated our tool-kit with the known inhibitor p5T, and we established a mini-screen to identify compounds that modulate this PPI in dose-response experiments. Finally, we discovered several compounds disrupting CDK5-p25 PPI, which had not been identified by other screening or structure-based methods before.

## Introduction

Protein-Protein interactions (PPIs) are central to most essential cellular mechanisms including gene expression, protein translocation, cell cycle progression and signal transduction. The cellular proteome is organized in often large, stable and transient complexes, which in humans can contain ten and more subunits, stabilized by a plethora of PPIs. Characterization of these interactions in their native cellular environment, and following their dynamic assembly and disassembly, is a vital prerequisite for understanding cellular mechanisms and their malfunction in disease states^1^.

Powerful methods have been developed for studying PPIs in a cellular context *in vivo*, including fluorescent resonance energy transfer (FRET), two-hybrid systems, bioluminescence resonance energy transfer (BRET), protein-fragment complementation assays and bimolecular fluorescence complementation (BiFC)^2^. In BiFC, two proteins or domains that are thought to interact are fused to the C-terminal and N-terminal halves, respectively, of a split fluorescent reporter protein^3^. Once the PPI between the interactors is formed, the two split parts of the reporter can reassemble thus restoring the fluorophore, resulting in a detectable fluorescent signal^3^. Reassembly does not occur spontaneously, but takes place only if the proteins or domains of interest are indeed interacting^4^. In contrast to other PPI analysis methods notably FRET, BiFC does not require previous knowledge of the three-dimensional structure of the complex studied, or demanding post-acquisition image processing for data interpretation. Furthermore, BiFC provides direct visualization of the PPI by a simple fluorescence microscope already at low expression levels due to the strong fluorescence of the fluorescent protein. On the other hand, FRET would allow real time detection of complex association and dissociation, while fluorophore formation is irreversible in a BiFC assay, ruling out detection of dissociation dynamics. Several other methods have been developed for PPI detection including BRET (Bioluminescent resonance energy transfer) imaging, or more recently NanoBiT (nano-luciferase binary technology, Promega) where two split parts of a reporter, luciferase, are fused to the interactors. An advantage of BiFC over other technologies is that it does not require exogenous agents which may generate toxicity or perturbations of cell function and metabolism. BiFC was successfully applied to visualize protein interactions in a range of cellular models^5–7^. Moreover, BiFC has been particularly useful to evaluate the effect of small molecules on protein complex formation in high throughput screens (HTS)^8,9^.

For efficient cell-based screening assays of PPIs by BiFC, at least two genes of interest need to be co-produced efficiently in a single cell. If complex multicomponent assemblies are studied, even more genes that encode for interactors will need to be expressed. Moreover, the individual expression levels must be ideally the same in each cell assayed in the experiment. Earlier, we had developed MultiMam^10^, a modular, plasmid-based expression system to co-produce multiple genes at defined ratios in mammalian cells, and we demonstrated its efficacy for multigene expression in transiently or stably transfected mammalian cells^11^. MultiMam relies on small synthetic progenitor DNA plasmid modules, that are assembled into a multigene expression construct containing multiple expression cassettes by a procedure called tandem recombineering (TR)^12^. TR exploits sequence and ligation independent cloning for gene insertion iteratively coupled to site specific recombination by the Cre-LoxP fusion reaction^12^. The unique modularity of MultiMam is optimally suited for setting up a highly efficient BiFC tool-box to assay PPIs of interest, by rapidly creating in parallel all interactor-fluorophore fusion pairs required to choose the optimal BiFC setup^13^.

Furthermore, we set out to make our toolbox readily adaptable to a wide range of cellular models, including cell types which are known to be recalcitrant to plasmid-based transfection. For this purpose, we outfitted our modified MultiMam-based BiFC system with elements required to insert the mammalian-active multigene expression construct into a baculovirus-based multigene delivery system, MultiBacMam. MultiBacMam is based on our original MultiBac baculovirus we had developed for multiprotein complex production in insect cells^14,15^. MultiBacMam is characterized by very large recombinant DNA cargo capacity, high mammalian cell transduction rates, low toxicity, safe handling and the provision to modulate protein expression levels by virus dosage. MultiBacMam can be applied efficiently to a wide range of mammalian cells lines^16^.

We applied our MultiBacMam BiFC tool-kit to analyze the PPI between CDK5 kinase and p25. CDK5 is a cyclin-dependent kinase that is involved in neuronal development and maintenance of the neuronal architecture and neurodegeneration. Its deregulation has been related to various neurodegenerative diseases such as Alzheimer’s disease, Parkinson’s disease, Huntington’s disease, ischemia and amyotrophic lateral sclerosis^17^. CDK5 activity relies on the binding to its activators p35 and p39. The cleavage of p35 by the protease calpain generates p25, which binds to CDK5 modifying the cellular localization of the complex, hyperactivating CDK5 and altering the recognition of its substrates. These modifications are implicated in neurotoxicity and thus in neurodegenerative diseases^18,19^. The CDK5-p25 PPI is a highly attractive pharmaceutical target. However, to our best knowledge, a successful mammalian cell-based screening strategy is markedly lacking to date. The first specific inhibitor of CDK5 described *in vitro* and *in vivo* is p5^20–23^, which represents a peptide fragment of p35. It was shown to inhibit the CDK5-p25 PPI and to rescue cortical neurons from induced apoptosis^22^. Further, in an Alzheimer’s mouse model, p5 rescued spatial working memory and motor deficits^23^.

We report here the first mammalian cell-based assay that integrates BiFC, our combinatorial MultiMam kit and the MultiBacMam baculovirus for highly efficient gene delivery. This toolbox can be used for plasmid-based transfection, baculorivus-mediated transduction and for stable cell line generation in a broad range of cellular models, by using the same set of reagents. We applied our MultiBacMam BiFC tool-kit to the CDK5-p25 interaction pair, thus establishing for the first time a screening against this interaction in a native-like, cellular context *in vivo*, in a set-up that is scalable to a *bona fide* HTS if desired. In our studies, we discovered three compounds which effectively abolished the CDK5-p25 PPI we analyzed.

## Results

### BiFC-Assay development, visualization of CDK5-p25 interaction

**Figure 1:**
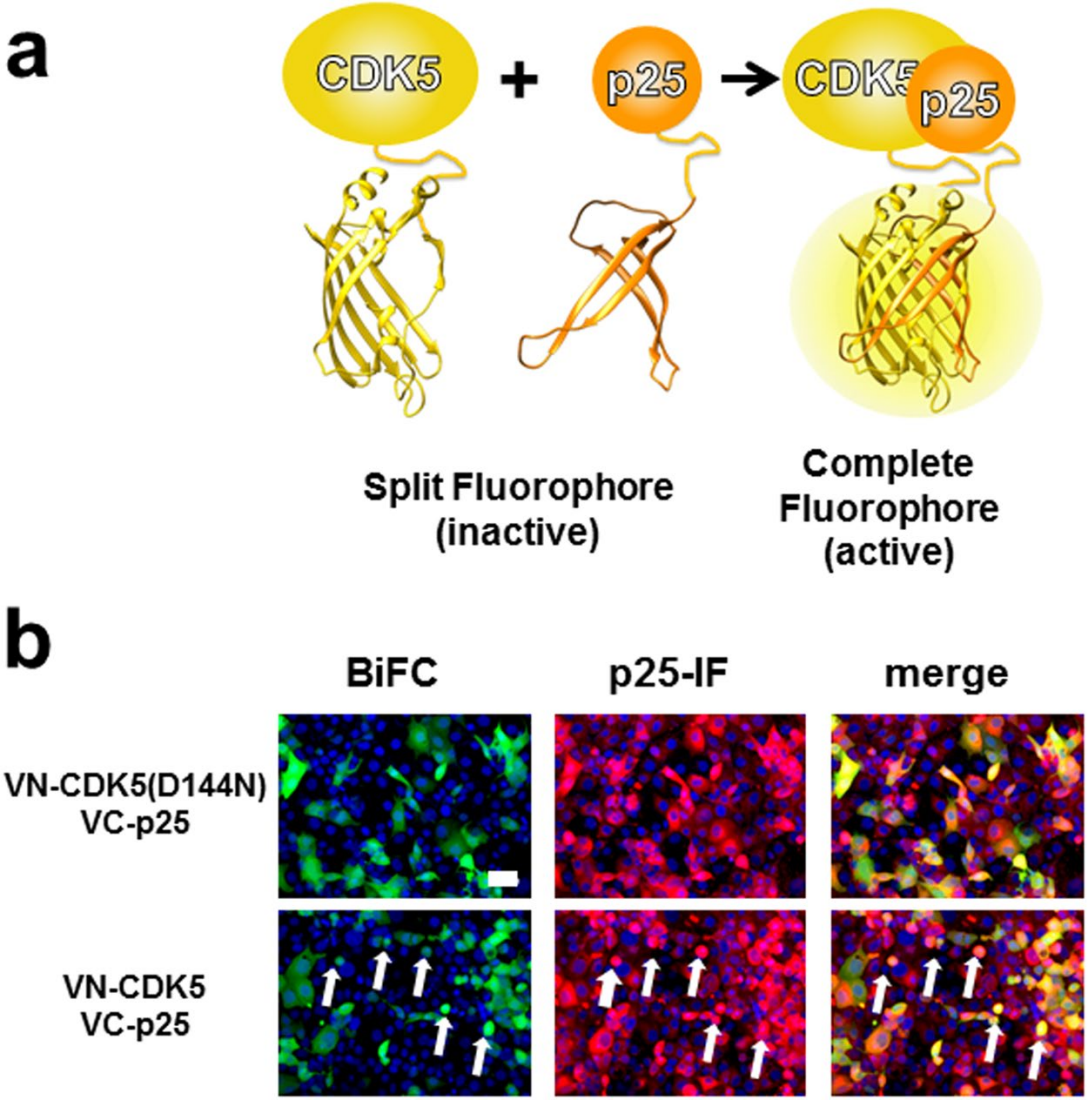
Bimolecular Fluorescence Complementation (BiFC), CDK5-p25 PPI. (**a**) Principle of BiFC analysis of CDK5-p25 PPI shown in a schematic view. Fragments of fluorescent protein Venus are fused to the proteins of interest (CDK5, p25). When CDK5 and p25 interact, active fluorophore is formed by reassembly of the fragments. (**b**) Cos7 cells transfected with VN-CDK5(D144N)/VC-p25 or VN-CDK5/VC-p25. Cells were fixed and scanned at 16 hours post-transfection. Immunofluorescence with anti-p25 antibody confirms expression of p25. Dead cells are marked (white arrows). Scale bar, 50 µm.

In order to set up an efficient BiFC assay, it is necessary to test the different combinations of bait and prey proteins, fused to the fluorescent protein fragments at their N or C terminal ends^13^. For each BiFC assay, this results in eight possible combinations for an interactor pair. We created a set of plasmid reagents for BiFC based on our MultiMam system^11^ (Table 1) comprising the DNA encoding the split fluorophore parts. Proteins of interest can be inserted by methods of choice (conventional cloning, sequence and ligation independent cloning methods) giving rise to N-terminal (Nt) or C-terminal (Ct) fusions. CDK5 and p25 were thus fused to the fragments of the split Venus fluorophores VN (amino acids 1-154) or VC (amino acids 155-258) (Fig. 1a) in plasmid modules pACEMam1 and pMCDP, respectively. In the MultiMam system, individual plasmid modules are recombined by Cre-LoxP fusion, ensuring expression of all proteins of choice in all transfected cells at defined ratios, yielding homogeneous cell populations^10^. Plasmids fused by Cre were transfected into COS7 cells to test the efficiency of the different combinations in BiFC. The highest number of positive cells (i.e. cells yielding detectable fluorescent signal) and fluorescence intensities were reached when both CDK5 and p25 were tagged on their N-termini (data not shown). The CDK5-p25 interaction can cause cell death by chromosome condensation^18^. To forestall widespread cell death in the transfected cell population, a mutation in the CDK5 catalytic domain (D144N) was introduced. CDK5D144N evidenced lower cell toxicity as compared to wild-type 16 hours after transfection (Fig. 1b). Based on these results, we selected the construct with split Venus fused to the N-termini of CDK5D144N and p25 for the experiments described below.

**Table 1:** BiFC plasmid reagents constructed in this study*.

Constructs tagged at N-term	Constructs tagged at C-term
pACEMam1-VenNt-GOI	pACEMam1-GOI-VenNt
pACEMam1-VenCt-GOI	pACEMam1-GOI-VenCt
pACEMam1-mCherry-GOI	pACEMam1-GOI-mCherry
pACEMam1-eGFP-GOI	pACEMam1-GOI-eGFP
pMDCP-VenNt-GOI	pMDCP-GOI-VenNt
pMDCP-VenCt-GOI	pMDCP-GOI-VenCt
pMDCP-eGFP-GOI	pMDCP-GOI-eGFP

*GOI,gene of interest; Ven, Venus fluorescent protein; Nt, N-terminal fragment, Ct, C-terminal fragment, pACEMAM1 and pMDC, plasmid reagents from MultiMam system^10^ (http://www.embl.fr/multibac/multiexpression_technologies/multimam).

### MultiBacMam gene delivery tool

**Figure 2:**
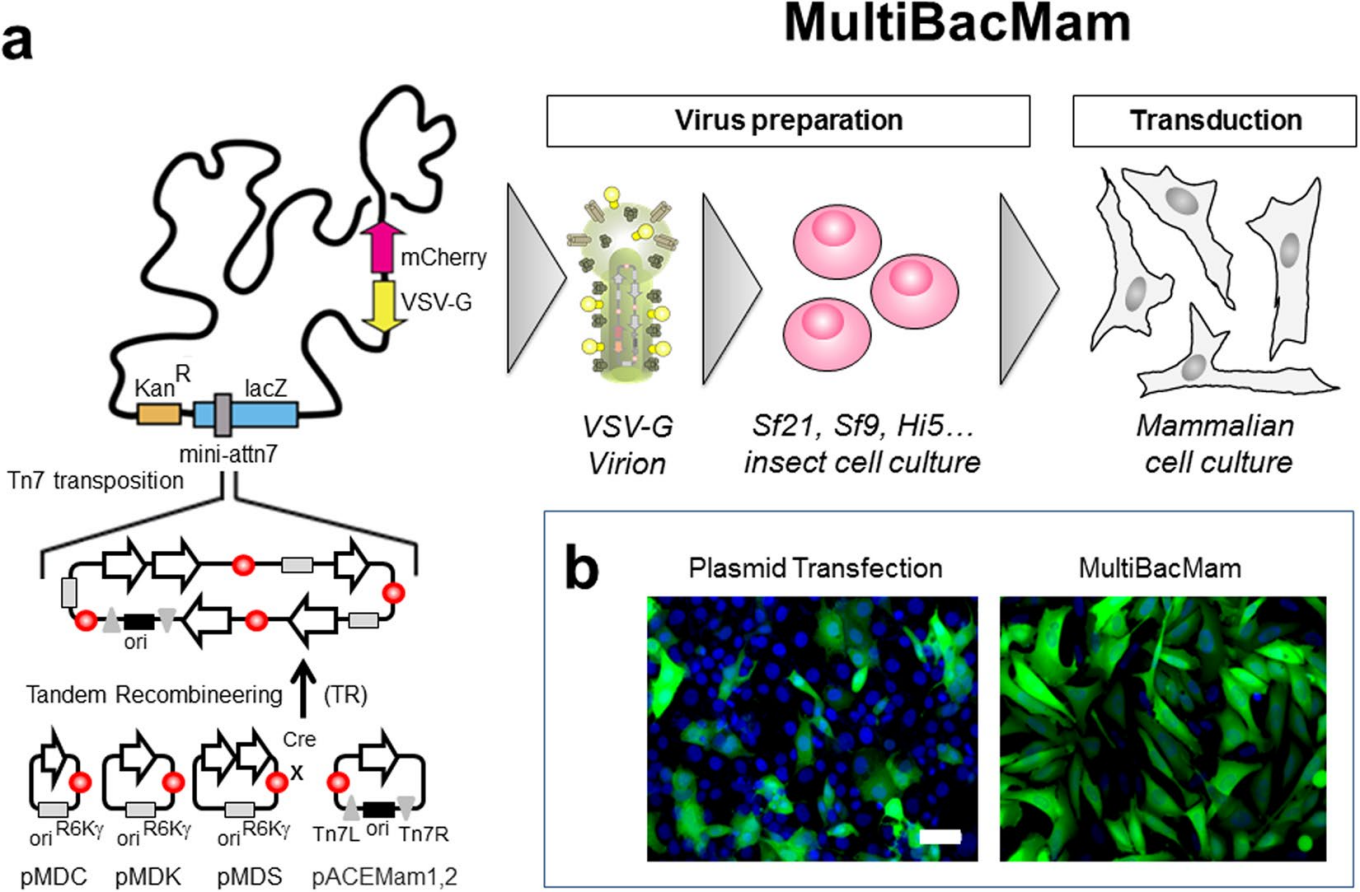
MultiBacMam baculovirus-mediated transduction. (**a**) MultiMam plasmid DNA modules (pACEMam1,2; pMDC, pMDK, pMDS)^10^ containing expression cassettes under mammalian-active promoter control are combined into plasmid fusions by using the tandem recombineering (TR) method, and then integrated into the MultiBacMam baculoviral genome by Tn7 mediated transposition. Tn7L and Tn7R denote short DNA segments present on pACEMam1 and pACEMam2 which are recognized by the transposase. Ori stands for a regular origin of replication derived from pBR322, ori^R6Kγ^ denotes a conditional replication origin depending on a pir^+^ background. The attachment site (mini-attn7) is embedded in a *LacZ* gene enabling blue-white screening of positive integrands in E. coli cells harboring the baculoviral genome as a bacterial artificial chromosome (BAC). Kan^R^ stands for kanamycin resistance marker. Sf21, Sf9 and Hi5 denote commonly used insect cell lines. MultiBacMam contains expression cassettes encoding for mCherry and vesicular stomatitis virus glycoprotein (VSV-G) integrated in the viral backbone. Composite MultiBacMam virus is used to infect insect cells for baculovirion production and amplification. VSV-G and mCherry are under baculoviral promoter control and thus expressed in insect cells. Virus performance can be followed by eye due to the red color caused by mCherry. High-quality MultiBacMam virus is then used to transduce mammalian cells. Virion image is adapted from drawing kindly provided by Kari Airenne^40^. (**b**) Efficacy of plasmid-based transfection compared to MultiBacMam-mediated transduction demonstrating the superior MultiBacMam approach. U2OS cells were transfected or transduced with the construct VN-CDK5(D144N)-VC-p25. BiFC signal is in green and Hoechst 33342 staining is shown in blue. Scale bar, 50 µm.

To expand the scope of our BiFC tool-kit to virtually every mammalian cellular model including cell lines recalcitrant to plasmid-based transfection, we next developed the MultiBacMam gene delivery tool, a modified baculovirus derived from our original MultiBac baculovuris/insect cell expression system. Baculoviruses have been shown to efficiently transfect, but not to replicate in, mammalian cells^24,25^. We had engineered the MultiBac baculoviral genome earlier to optimize multiprotein expression in insect cells by removing detrimental functionalities from the genome^26^. Our MultiBacMam baculovirus retains these advantages which include delayed lysis of cells and reduced proteolysis, resulting in high quality baculovirion production in infected insect cells^16^. We modified the MultiBac genome by integrating an expression cassette containing the gene encoding for vesicular stomatitis virus glycoprotein (VSV-G), which was shown to enhance mammalian cell transduction^27^. Furthermore, we co-integrated a baculoviral promoter-driven mCherry fluorescent protein expression cassette to allow monitoring of virus amplification in infected insect cell cultures by observing red color (Fig. 2a). The pACEMam derived BiFC plasmid modules contain short DNA sequences (Tn7L and Tn7R) for T7 transposase-mediated integration into a mini-attn7 attachment site present on the MultibacMam baculoviral genome (Fig. 2a). A fused construct comprising the functional VN-CDK5(D144N)/VC-p25 BiFC pair was integrated by Tn7 transposition into the MultiBacMam genome (Fig. 2a) in *E.coli* cells (DH10MultiBacMam) harboring the viral genome as a bacterial artificial chromosome and a helper plasmid producing the Tn7 transposase. Positive clones were selected and recombinant baculovirus produced following established protocols^28,29^. Composite MultiBacMam baculovirus produced in insect cells was then used to transduce U2OS, HeLa and Cos7 cells lines at a multiplicity of infection (MOI) ranging from 25 to 500. BiFC signal was observed in all cell lines tested. In HeLa and Cos7, a MOI of 200 was required to visualize the interaction. In U2OS, in contrast, BiFC could be observed already at 50 MOI, although a larger fraction of positive cells and higher fluorescence intensities could be obtained at 500 MOI (data not shown). Transduction rates and expression levels were significantly higher using MultiBacMam as compared to plasmid-based transfection (Fig. 2b). We also observed that MultiBacMam induces expression of exogenous constructs early, thus enabling us to carry out transduction and also our assay within one day.

We performed a time course assay to determine optimal assay kinetics. A time-lapse scan was carried out during 20 hours post transduction monitoring the number of positive cells and Venus intensity values. Maximum number of positive cells was reached seven to eight hours after transduction (Supplementary Figure S1A). The decrease of the fraction of positive cells that we observed can probably be attributed to residual cytotoxicity triggered by the CDK5D144N-p25 interaction in spite of using the mutant (Supplementary Figure S1C). Venus intensity values continuously increased, probably due to the irreversible reconstitution of the split fluorophore (Supplementary Figure S1B). We scanned plates in our experiments at 16 hours after expression, when maximum number of positive cells and high levels of intensities were observed.

### Reference inhibitors

**Figure 3:**
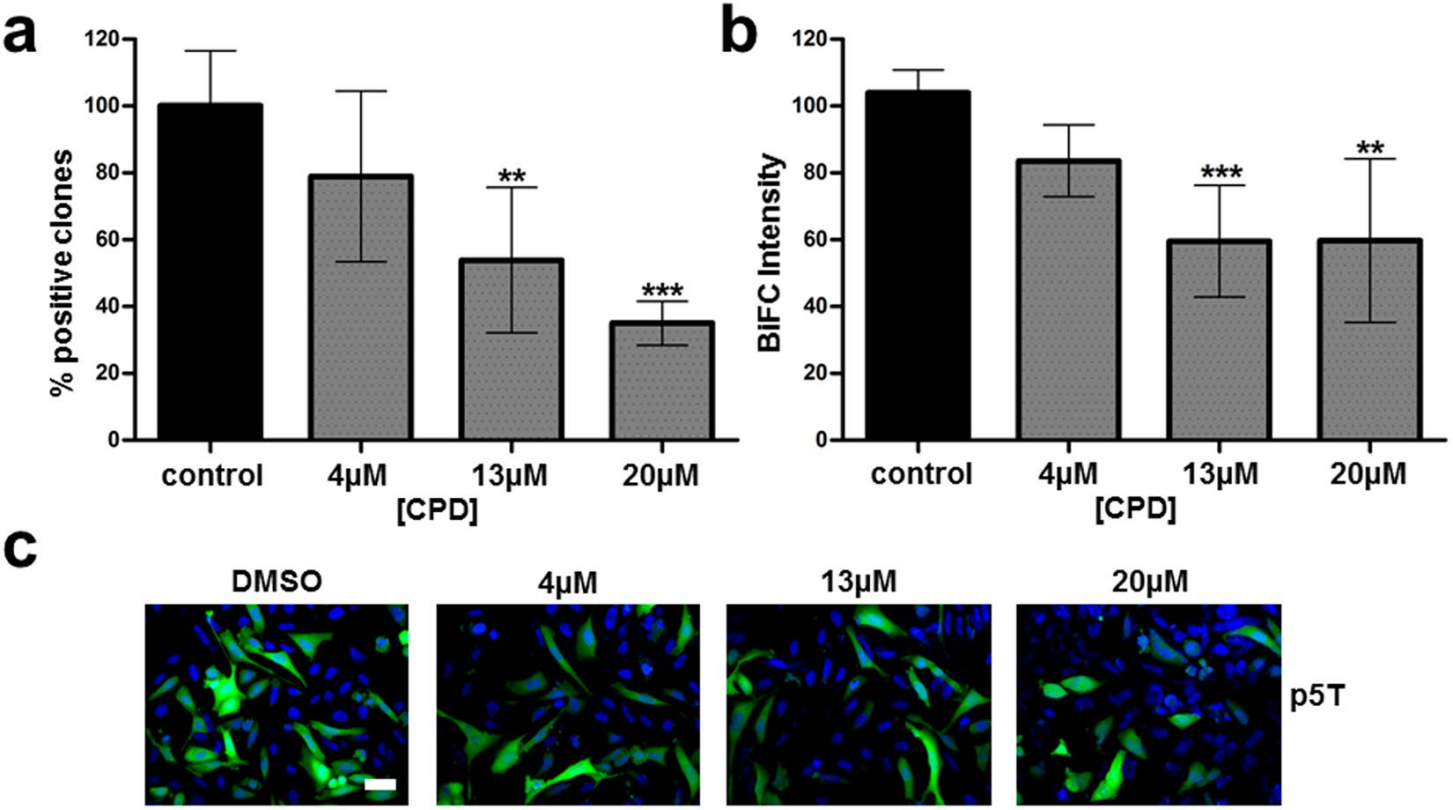
CDK5-p25 PPI inhibitor p5T. U2OS cells transduced with a MultiBacMam virus expressing VN-CDK5(D144N)/VC-p25 were treated with p5T for 16 hours. The percentage of positive cells (**a**) and fluorescence intensity (**b**) were recorded. Control results are plotted in black Results with p5T peptide are represented in dark gray with dots. Mean values and standard deviations from three independent experiments are shown. P values were obtained using on-way ANOVA test. Significance levels are P < 0.01 (**), P < 0.001 (***). (**c**) U2OS cells were transduced with the construct VN-CDK5(D144N)/VC-p25 and treated with 4, 13 and 20 µM compound. BiFC signal is shown in green and Hoechst 33342 staining is shown in blue. Scale bar, 50 µm.

A first confirmed inhibitor is the p5T peptide, derived from p25^20,21,23^. U2OS cells were transduced with the MultiBacMam BiFC virus expressing VN-CDK5/VC-p25 (see Material and Methods). Fluorophore formation is irreversible^3,30^ and the Venus signal can be stable for several hours (Supplementary Figure S1A and B). Therefore, we decided to add the peptide after removal of the viral supernatant in order to increase the assay window. After 16 hours treatment with different p5T concentrations, cells were fixed, nuclear stained and scanned. Results were expressed as percentage of positive cells normalized to a control (transduced cells treated with DMSO, no peptide added). At high concentrations of p5T we observed a reduction of around 60% of BiFC positive cells, with a 40% drop in cell BiFC intensity as compared to the control (Fig. 3c). Concentrations above 13 µM resulted in a significant reduction of both, positive cells and fluorescence intensity (Fig. 3a,b). At a peptide concentration of 20 µM, minor cytotoxicity was observed which did however not account for the reduction in the BiFC signal (Supplementary Figure S2A). In order to verify the specificity of p5T inhibition, the peptide was counter tested in two additional BiFC PPI assays. As shown in Supplementary Figure S3, p5T peptide had no inhibitory effect neither on MLKL-RIPK3 protein PPI and nor on Tau dimerization. We conclude from these experiments that the results obtained with p5T compellingly validate the utility of our MultiBacMam BiFC kit as a tool to identify modulators of the CDk5-p25 PPI.

### Small molecule compound screening

Next, we adapted our MultiBacMam BiFC tool to automation and high-throughput in a 384 wells microplate format as a proof-of-concept for future HTS applications. A set of diverse structures was selected to yield the final number of 35 compounds for a pilot screening.

**Figure 4:**
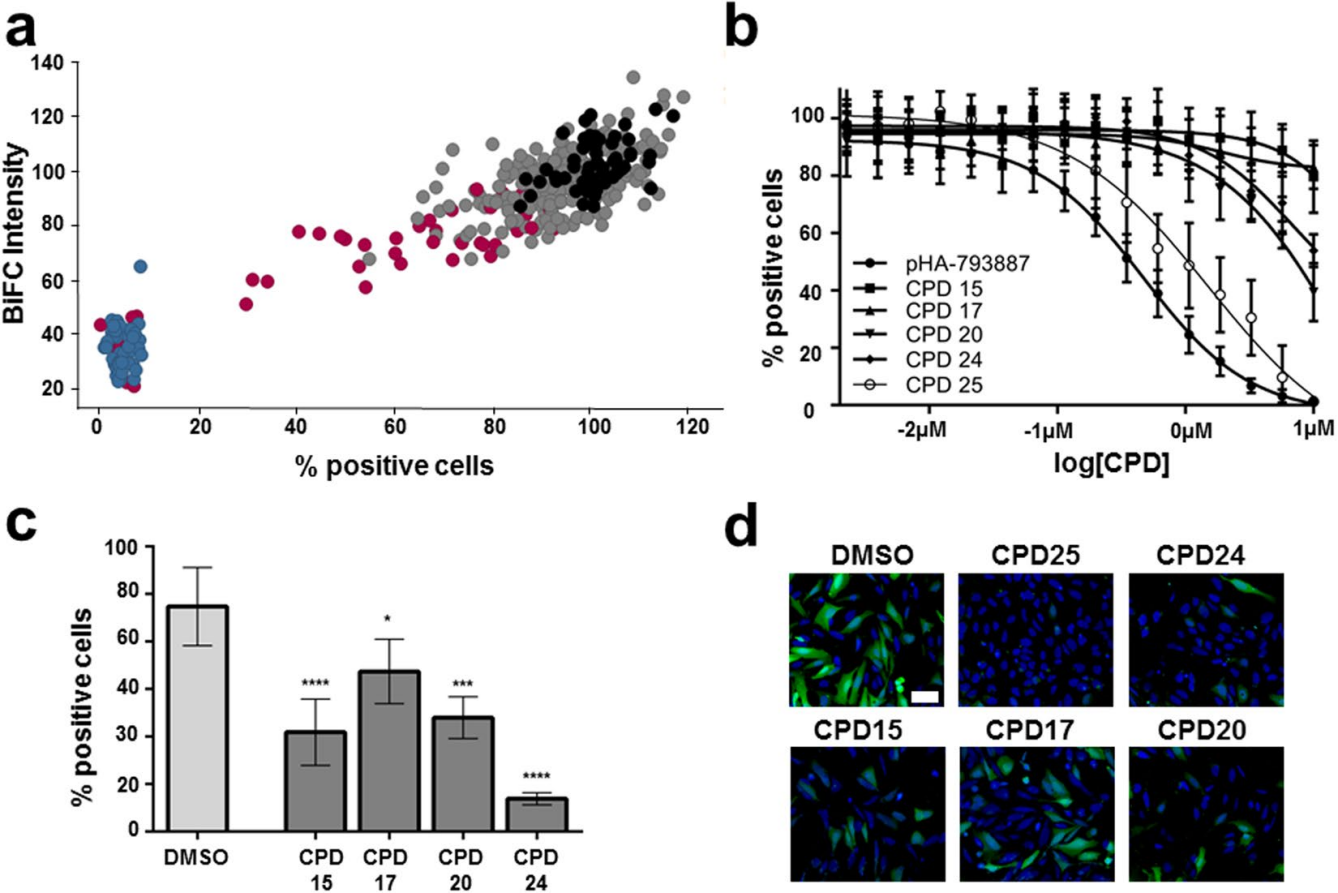
MultiBacMam BiFC small molecule compound screening. (**a**) BiFC intensity versus percentage of positive cells obtained after 16 h treatment with 10 µM of each compound (grey dots) is shown. Values were normalized to 100% of the DMSO control (black dots). pHA-793887 values are colored in blue. Hits from the screen are plotted in red. Eight replicates for each compound were made. (**b**) Dose-response curve of compound CDP25 and CDP24 is shown. Mean values and SDs from eight experiments are shown. (**c**) U2OS cells were transduced with VN-CDK5(D144N)/VC-p25 and treated with 10 µM of CPD25 and CDP24. BiFC signal is in green and Hoechst 33342 staining is in blue. Scale bar, 50 µm. Percentage of positive cells obtained after 16 hours treatment with 50 µM compound each. Mean and SD of three experiments are shown. P values were obtained using on-way ANOVA test. Significance are P < 0.1 (*), P < 0.01 (**), P < 0.001 (***) and P > 0.0001 (****). (**d**) U2OS cells transduced with the construct VN-CDK5(D144N)/VC-p25 and treated with 50 µM of compound. BiFC signal is in green and Hoechst 33342 staining is shown in blue. Scale bar, 50 µm.

Each compound was tested in two independent screens with 16 data point dose-responses ranging from 0.002 to 10 µM. Results were normalized to the average value of control cells treated with DMSO (see Materials and Methods). In our screen, five compounds (Table 2) exhibited a reproducible effect and were selected as primary hits for further studies (Fig. 4). In order to confirm the inhibition and to obtain the largest effect, these compounds were retested at a higher concentration (50 µM). Compound CPD25 was the most potent hit reducing the percentage of positive cells by more than 90% and yielding an IC_50_ of 1.39 µM. Compound CPD24 showed a decrease of 55% as compared to control at 10 uM (Fig. 4b), and at 50 µM the decrease in positive cells reached 85% (Fig. 4c). Both CDP15 and CDP20 showed a 50% decrease in the percentage of positive cells whereas CDP17 only inhibited 30% at 50 uM. (Fig. 4c,d).

**Table 2:** CDK5-p25 PPI active small molecule compounds identified in this study.

Compound Nr.	Roche Indentifier	Chemical formula
CPD15	RO5056904	
CPD17	RO5089465	
CPD20	RO5223732	
CPD24	RO5258638	
CPD25	RO5454291

Next, the confirmed compounds were counter screened in the MLKL-RIPK3 and Tau BiFC assays (Supplementary Figure S3). Similarly to pHA-793887, CPD25 proved to be a non-specific fluorescence complementation inhibitor as it triggered an almost complete loss of BiFC signal at 10 μM. As in CDK5-p25 assay, one digit micromolar IC_50_ were obtained for both counter assays (5.84 ± 0.05 μM and 3.21 ± 0.05 μM for MLKL-RIPK3 and Tau assays respectively), illustrating the non-specific inhibitory effect of the compound on unrelated PPI pairs. As shown on Supplementary Figure S4, CPD25 did not display any quenching activity on EGFP fluorescence. The unspecific effect observed on our BiFC assay might thus be attributed to the disruption of Venus fragments complementation. For CPD24, we obtained a reduction of 16 and 20 points in the percentage of MLKL-RIPK3 and Tau BiFC positive cells respectively only at 10 μM. This unspecific BIFC blockade occurs at higher dose (~10-fold) and displays a much low effect size compared to the effect that was observed on CDK5-p25 PPI (Fig. 4). CPD15, 17, and CPD20 were unable to disrupt neither MLKL-RIPK3 interaction nor Tau dimerization even at high concentration demonstrating the specificity of the inhibition seen on CDK5-p25.

## Discussion

Here we present our MultiBacMam BiFC toolbox, a versatile new platform that integrates the advantages of BiFC with the flexible modularity of our MultiMam system and the highly efficient MultiBacMam baculovirus transduction reagent in a setup compatible with automation and HTS. The percentage of positive cells, the (lack of) toxicity of the overexpressed target proteins, and obtaining a reasonably wide assay window are critically important parameters for obtaining robust screening outcomes and key to success in assay development. The MultibacMam BiFC toolbox we present here combines numerous advantages beyond the current state-of-the-art^31–34^. Our toolbox relies on accessible synthetic plasmid DNA modules which are flexibly combined into multiple gene expression cassettes by our TR protocol, enabling rapid identification of combinations best suited for BiFC. The resulting multi-expression construct is a single reagent comprising the PPI pairs of interest, and its administration ensures that all components are expressed at the same level in every single cell, resulting in hitherto unattained homogeneity and reproducibility of the assay experiments^11^. Moreover, BiFC constructs generated by the TR method can be introduced into MultiBacMam, our engineered baculoviral genome by Tn7-based transposition. MultiBacMam has been optimized for baculovirion production in insect cells, yielding high quality baculovirus suitable for mammalian cell transduction (Fig. 1b). A low number of positive cells and a high variability that often are found in cellular assays using current technology can be detrimental for the development of efficient screening assays. Our MultiBacMam BiFC tool-kit, in contrast to existing system, has the benefit of increasing the number of positive cells (Fig. 2c). Moreover, it markedly reduces the variability between individual wells as well as between independent experiments. The system we developed enables plasmid-based transient transfection, highly efficient baculovirus-mediated transduction, and even stable cell line generation with the very same set of reagents and can be easily adapted to the particular necessities of the PPIs analyzed, optionally in high throughput. Moreover, our system is not limited to the analysis of one BiFC pair only – the very large foreign DNA cargo capacity of the MultiBacMam viral genome enables concomitant study of a number of BiFC pairs, one each for every PPI of interest, only limited by the number of split fluorophores utilized in parallel for detection. Thus, entire signaling cascades or metabolic pathways, or a multitude of interactions within a complex of interest and its dynamics, can be studied simultaneously using our approach.

We applied our Multibacmam BiFC toolbox successfully to assay the CDK5-p25 PPI using mammalian cells, more accurately representing their native-like environment. In our assay, exogenous substrates are not required for detecting the PPI under investigation, greatly simplifying the experimental set-up and largely eliminating possible artefacts due to permeability or toxicity of the substrates added to cells.

A possible concern about the use of a cell-based BiFC system for screening may be the irreversible formation of the fluorophore. Ideally, therefore, the PPI needs to be challenged with the compounds screened before the fluorophore is irreversibly formed. Time course experiments (Supplementary Figure S1) evidenced that there is a window of several hours between the transfection or transduction and the irreversible formation of Venus fluorescent protein. We showed that this can be exploited advantageously by adding the compound directly after transfection, thus blocking the PPI. On the other hand, evidence exists that the CDK5-p25 PPI is difficult to disrupt once it has been formed^35^. The inhibitory effect of 3α-amino-5α-androstane was only detected over the preformed CDK5-p25 complex. The strength of the PPI, and the irreversible nature of fluorophore formation had to be taken in account in the design of the assay. Therefore, we removed the viral supernatant shortly after transducing the cells, and replaced with media containing the compounds, thereby ensuring that the compound is present prior the formation of the CDK5-p25 PPI. In this way compounds have the opportunity to inhibit the interaction, consequently reducing the BiFC signal. As a proof-of-concept, we visualized the previously established effect of p5T in CDK5-p25 PPI inhibition (Fig. 2 and Supplementary Figure S3), compellingly validating our approach. An inhibitory effect of 3α-amino-5α-androstane was likewise reported before^22,35^. However, our assay did not reproduce this published data. We note that the previous report described a yeast-based assay where two lysates containing overexpressed proteins were mixed. In contrast, we use here a mammalian cell-based assay. On the one hand, in our approach, the capacity of the compound to penetrate cells may be an issue. On the other hand, the native cellular environment could impact on the efficiency of the compound to find and bind p25. Moreover, we note a toxic effect on the cells with this compound, which prohibited increasing the compound concentration to above 13 µM (Supplementary Figure S2A).

We then adapted our approach to HCS in a 384-well format, to screen a selected set of small molecule compounds from a Roche internal library and we could identify further modulators that were able to partly or even completely disrupt the CDK5-p25 PPI (Fig. 4). Counter assays with alternate BiFC pairs allowed filtering out compounds displaying unspecific complementation blockade or fluorescence quenching.

In summary, we present here a versatile, robust and efficient novel toolbox for studying PPIs by BiFC in a native-like mammalian cellular context and we have successfully applied it in a screening experiment to identify potential hits that disrupt the CDK5-p25 PPI. By using our toolbox, we have established here the first screening flow in mammalian cells targeting the CDK5-p25 PPI and revealed further small molecule drug candidates inhibiting CDK5-p25 PPI, setting the stage for future HTS assays. We anticipate that our MultiBacMam BiFC toolbox will be successfully applied to study many PPIs in a wide range of cell types in native-like environments, to identify powerful and efficient modulators of PPIs including those implicated in human disease.

## Methods

### Generation of BIFC constructs

pACEMam1 and pMDC vectors from the MultiMam system were used as a backbone for the BiFC system^11^. pMDC was modified adding an expression cassette carrying puromycin as a mammalian selection marker by SLIC^36^. The Puromycin expression cassette (EF1a-Puromycin-PolyA) was obtained by PCR using the following primers:

5′TGCTTCCGGTAGTCAATAAAAAGGATCTGCGATCGCTCCGGTGCCCG3′ (Puro-S)

5′GTCTATTGCTGGTTTACCGGCGCGCCGCACACAAAAACCAACACACAG3′ (Puro-AS).

A SHANK3 loxP-EF1a-Puro-loxP cassette was used as a template. pMDC was linearized by AgeI digestion. SLIC for gene insertion was carried out as previously described^36^. Nt Venus (1–154) and Ct-Venus (155–238) were fused to indicate interaction partners using RSIAT and RPACKIPNDLKQKVMNH linkers^37^ respectively.

In a first step, Venus protein fragments were fused t the N- and C- terminal end of each interaction partner and all eight possible combinations were tested for complementation (Fig. 1 and data not shown). For each PPI pairs, the optimal combination is indicated in Table 3. Full length mCherry and GFP were fused with CDK5 and p25 with a linker of 5 glycines. All constructs were synthetized by GenScript. Fusion proteins were then cloned by BamHI and XbaI RE in pACEMam1 and pMDCP plasmids.

**Table 3:** Location of split flourophores in PPI constructs studied.

Nt Venus (1–154)		Ct-Venus (155–238)	
Interaction partner	Fusion site	Interaction partner	Fusion site
CDK5	N-term	p25	N –term
MLKL	N-term	RIPK3	C-term
TAU	C-term	Tau	C-term

### Cell culture

Sf9 cells were cultured in suspension growth in Sf900II medium SFM (Gibco) in a 27 °C incubator. Cos7 were grown in DMEM (Gibco) containing 10% of fetal bovine serum (Gibco), 100 units ml^−1^ penicillin and 100 ug ml^−1^ streptomycin (Gibco). U2OS were grown in McCoy’s 5 A medium (Gibco) containing 15% of fetal bovine serum (Gibco) 100 units ml^−1^ penicillin and 100 ug ml^−1^ streptomycin (Gibco). Both cell lines were maintained at 37 °C with 5% of CO_2_. Cells were transfected with Xtreme gene HP DNA transfection Reagent (Roche) following manual instructions.

### MultiBacMam virus generation

MultiMam vectors carrying VN-CDK5 and VC-p25 constructs were inserted in DH10MultiBacMa cells by Tn7 transposition/blue white screening according to standard protocols^28^. Purified baculoviral genomes were transfected in Sf9 cells to prepare MultiBacMam virus were produced as previously described for another baculovirus^29^.

### BIFC Assay

Experiments were carried out in sterile, black clear-bottom 384 well plates (Cell carrier™ Perkin Elmer). U2OS cells were seed at density of cells 5 × 10^4^ cells/well. After 3 hours, attached cells were transduced with 20 µl/well of viral stock dilution in PBS (Gibco) at multiplicity of infection (MOI) of 500 MOI. After were centrifuged during 30′ at 1500 rpm. Then plates were incubated at 37 °C during 5 hours. Thereafter the virus solution was replaced with 40 ul of complete growth medium containing 3 mM sodium butyrate (ABCR) with or without compound depending of the experiment. Cells were incubated for 16 hours at 37 °C with 5% of CO_2_. Finally plates were fixed with 4% paraformaldehyde (PFA) and stained with 5 µM Hoechst 33342 (Sigma-Aldrich). After 10 minutes of incubation at room temperature, plates were washed with PBS (GiBCO) and stored at 4 °C until imaging. For the immunostaining against p25 cells were permeabilized and stained with PBS, BSA 0.2% and Triton-X 100 0.5% and p35 (C-19) antibody from Santa Cruz Biotechnology (diluted 1:100 and incubated at 4 °C o/n). Then, the plates were incubated for 2 hrs at 37 °C with a donkey anti-rabbit conjugated with the alexa fluor 647 secondary antibody (Invitrogen). Finally plates were washed with PBS and stored at 4 °C until imaging.

### Compounds

p5T correspond to the residues K254-A277 from p25 sequence and it was fused in the C-terminal a transactivator of transcription^23^. p5T (chemically synthesized by CS Bio co) and pHA-793887 (Selleck) were prepared diluting them in DMSO (10 mM). 3α-amino-5α-androstane was synthesized in house. The 35 compounds used in the mini screen were from a Roche internal library. All compounds were pre-dissolved in DMSO, diluted to 5 mM stock solutions. All compound stocks were stored at −20 °C until the day of the assay.

### Small compound screening

The 35 compounds were divided in two 384-well plates; DMSO curve was included as a control. Dose response curves were prepared performing serial dilutions (20/35) in DMSO starting in the first row at 200 µM. Plates were stored at −20 °C until the day of the assay. The assay day eight 384-well plates were seeded by a multidrop 384 system (Titerlek) with 5000 U2OS cells/well (4 replicates/compound plate). After 3hrs of incubation at 37 °C, plates were transduced with CDK5(D144N)/p25 virus as described above. A 1/20 dilution from the compound plate in McCoy’s media with 3 mM sodium butyrate was performed. After discard the virus solution, 40 µl of the previous dilution were transferred to each well. Plates were incubated 37 °C during 16 hrs. Finally plates were fixed with 4% paraformaldehyde (PFA) and stained with 5 µM Hoechst 33342 (Sigma-Aldrich). After 10 minutes of incubation at room temperature, plates were washed with PBS (GiBCO) and stored at 4 °C until imaging.

### High content imaging analysis

Cells were transduced or transfected and fixed as described above. Cells were stored in PBS until automated fluorescence microscopy analysis with Opera. Fluorescence images were acquired using the Opera QEHS reader (Perkin-Elmer) using a 20x objective. Hoechst stain was imaged with laser excitation at 405 nm and a 455/70 nm band-path filter (BP) for emission. The BiFC signal was imaged using laser excitation at 488 nm, and a 543/22 nm BP filter for emission and the immunostaining against p25 using laser excitation at 635 nm and 690/50 nm BP for emission.

### Absorbance spectrum and EGFP quenching assay

Compounds were diluted at 100 uM in PBS and the wavelength of maximum absorption at 26 °C was measured on an Enspire plate reader from Perkin Elmer. In order to determine effect of compounds on EGFP fluorescence, 150 ng of recombinant enhanced Green Fluorescent Protein (EGFP) were diluted in PBS and incubated for 4 h at Room temperature with indicated amounts of compounds. EGFP fluorescence was measured on an Enspire plate reader from Perkin Elmer (Ex_480 nm_/Em_510 nm_)^38,39^.

## Electronic supplementary material

Supplementary Figures
