# Supplementary material for: MultiBacMam Bimolecular Fluorescence Complementation (BiFC) tool-kit identifies new small-molecule inhibitors of the CDK5-p25 protein-protein interaction (PPI)
Source: Sci Rep. 2018 Mar 23;8:5083. doi: 10.1038/s41598-018-23516-x (PMC5865166; doi:10.1038/s41598-018-23516-x)
Supplement: Supplementary file 1 — Supplementary Figures [file 41598_2018_23516_MOESM1_ESM.docx]

Supplementary Figures

**MultiBacMam Bimolecular Fluorescence Complementation (BiFC) tool-kit identifies new small-molecule inhibitors of the CDK5-p25 protein-protein interaction (PPI)**

Itxaso Bellón-Echeverría^1,2^, Jean-Philippe Carralot^1^, Andrea Araujo Del Rosario^1^, Stephanie Kueng^1^, Harald Mauser^3^, Georg Schmid^1^, Ralf Thoma^1*^ and Imre Berger^4#^

^1^ Chemical Biology, pRED Innovation Center Basel, F. Hoffmann-La Roche Ltd, Switzerland.

^2^ European Molecular Biology Laboratory, Grenoble Outstation, 6 rue Jules Horowitz, 38042 Grenoble, France and Unit for Virus Host-Cell Interactions, Univ. Grenoble Alpes-EMBL-CNRS, 6 rue Jules Horowitz, 38042 Grenoble, France.

^3^ Global Technical Development, F. Hoffmann-La Roche Ltd., Switzerland

^4^ School of Biochemistry, Bristol University, Clifton BS8 1TD, United Kingdom

^#^ Materials & Correspondence:

Ralf Thoma ([ralf.thoma@roche.com](mailto:ralf.thoma@roche.com)); Imre Berger (imre.berger@bristol.ac.uk)

**Supplementary Figure S1:**

**
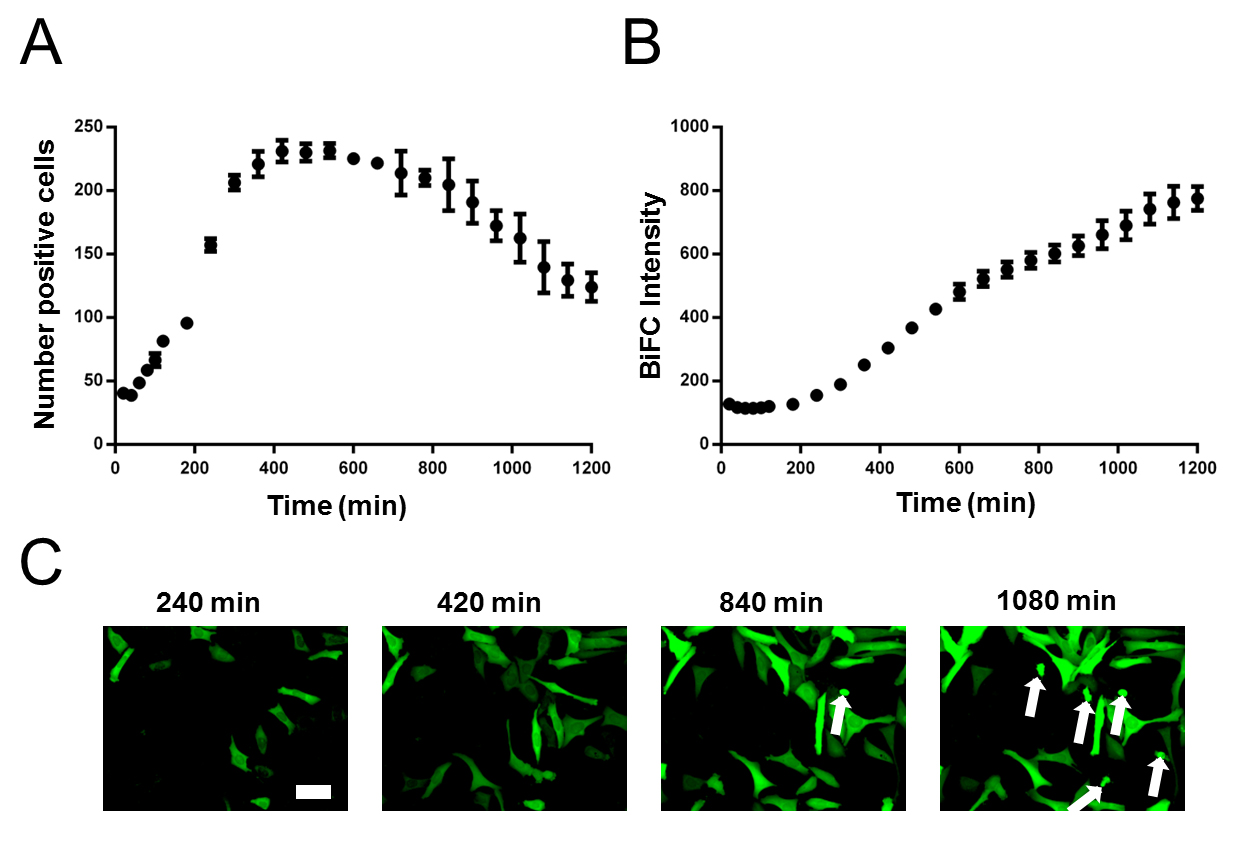
**

**Supplementary Figure S1. Time course, BiFC experiment**. U2OS cells transduced with VN-CDK5(D144N)/VC-p25 virus. Plates were scanned each half an hour during 20hours. **A**. Number of positive cells is shown. **B**. BiFC intensity values are shown. Mean values and SDs are from three independent experiments. **C**. Images of time points (min) 240, 420, 840 and 1080. BiFC signal is shown in green. Dead cells are marked (white arrows). Scale bar, 50µm.

**Supplementary Figure S2:**

**
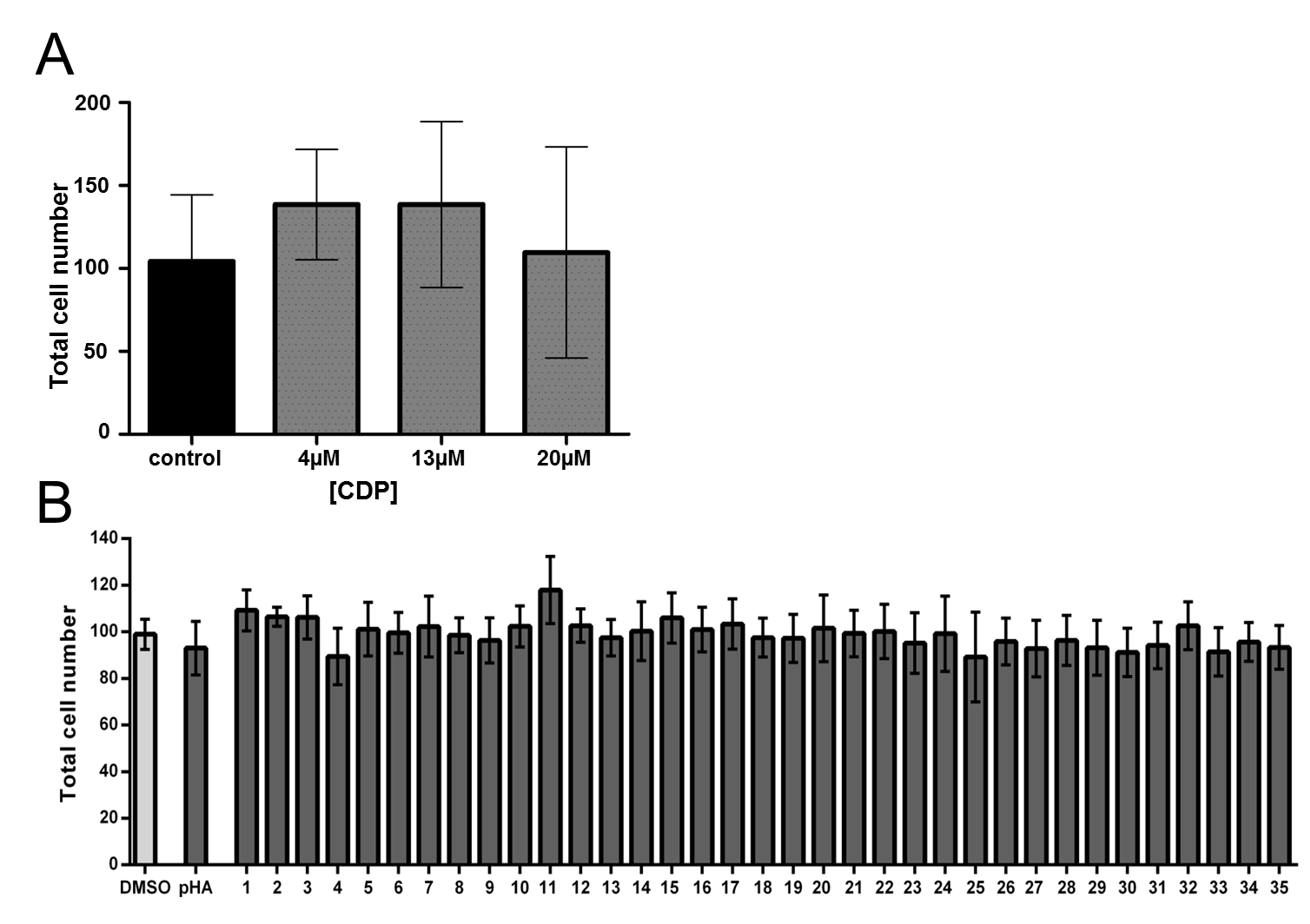
**

**Supplementary Figure S2.** **Total cell number in BiFC experiments**. **A**. The total cell number of the p5T (grey bars) is shown. **B**. Total cell number of the mini-screen is provided. Mean values and SDs are from three independent experiments.

**Supplementary Figure S3:**

**
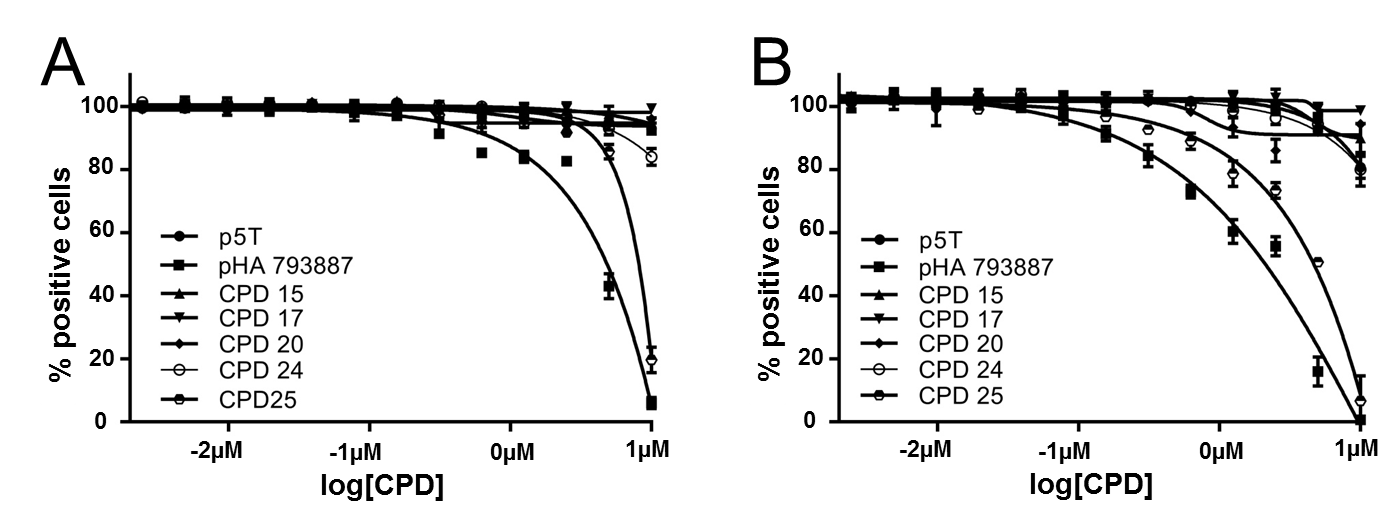
**

**Supplementary Figure S3.** U2Os cells were U2OS cells were transduced with MultiBacMam BiFC virus expressing VN-MLKL/VC-RIPK3 (**A**) and VN-TAU/VC-TAU (**B**). Then cells were treated with indicated amounts of compounds for 16h and percentage of BiFC positive cells was determined by High Content image analysis.

**Supplementary Figure S4:**


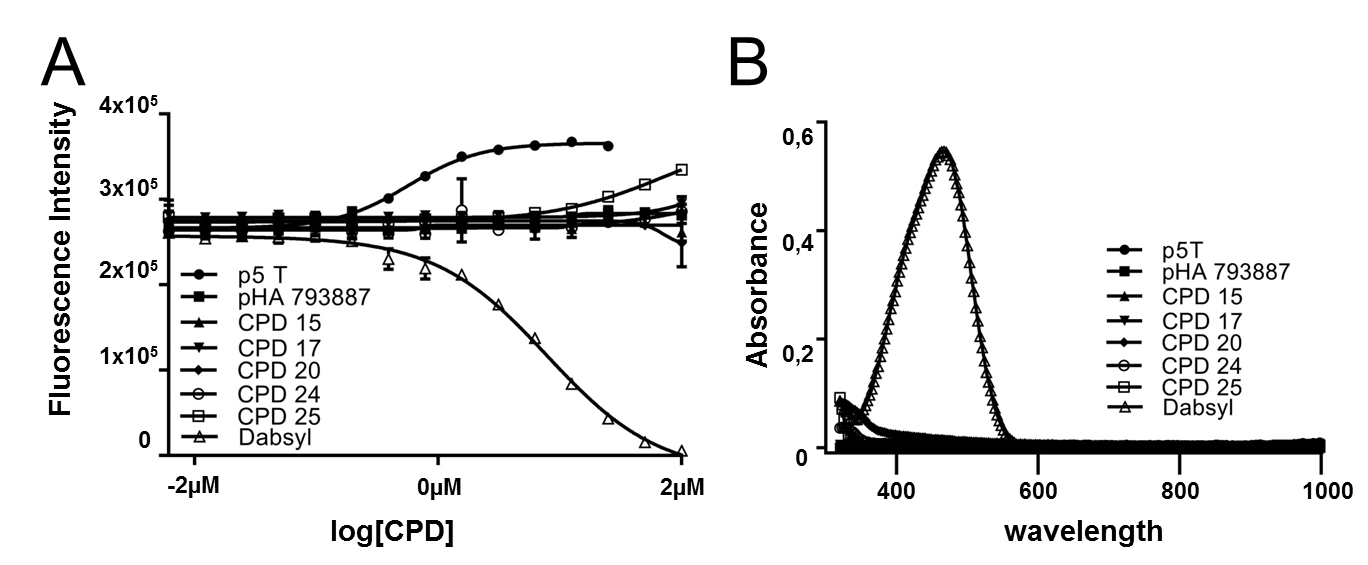


**Supplementary Figure S4.** **A.** Absorbance spectrum of compounds. Compounds were diluted in PBS at 26°C and absorbance we determined on a plate reader. **B.** Compounds were incubated with 150ng of EGFP during 4h at Room Temperature and Ex480nm/Em510nm was measured on a microplate reader ((Ex_480nm_/ Em_510nm_))
